# Technological solutions to capture data for patient reported outcomes using mobile devices

**DOI:** 10.1186/1745-6215-16-S2-P38

**Published:** 2015-11-16

**Authors:** Dionne Russell, Jonathan Gibb, Sharon Kean

**Affiliations:** 1 University of Glasgow, Glasgow, UK

## Background

Tools to capture patient reported outcomes within a clinical trials context have been evolving from paper based systems to web based data capture systems and now, more recently, using mobile technology. Recording patient reported outcome measurements (PROMs) using online or mobile devices can provide more accurate data by providing functionality such as flagging missing responses real-time.

However, even with the availability of good quality low cost mobile devices and the desire to complete PROMS using these devices it is often not feasible or desirable to provide a mobile device to each participant but instead to share a device among many.

## Methods

There are a number of design decisions that need to be considered when designing a mobile application for a device that will be shared between multiple participants - especially when having to deal with offline scenarios. In an offline system scenario, data has to be stored on the device and the application flow must not allow the current participant to access any other participants’ data. Security is critical and robust methods of ensuring the data are synchronised to a central database must be simple and effective. We will discuss the options and technical methods employed to provide these key processes.

## Conclusion

Based on lessons learned supporting recent studies which required mobile devices to be shared between multiple participants, we can now show what essential settings and device configuration have to be considered. In addition, we can conclude with application design methods provide successful solutions.

